# The Development and Evaluation of a Structured Continuing Professional Development Programme for Pharmacists in Kuwait: A Feasibility Study

**DOI:** 10.3390/pharmacy8040207

**Published:** 2020-11-05

**Authors:** Asmaa Al-Haqan, Shahad Al-Baghli, Al-Bandari Al-Enizi, Hailah Al-Dosari, Salah Waheedi

**Affiliations:** 1 Department of Pharmacy Practice, Faculty of Pharmacy, Kuwait University, Safat 13110, Kuwait; asmaa.alhaqan@ku.edu.kw (A.A.-H.); shahadbaghli@gmail.com (S.A.-B.); albandari.enezi@HSC.EDU.KW (A.-B.A.-E.); 2 Kuwait Ministry of Health, Mubarak Al-Kabeer Hospital, Jabriya 046300, Kuwait; hailahb995@gmail.com

**Keywords:** continuing professional development, competency development, workforce development, foundation training, pharmacy, Kuwait

## Abstract

Continuous education and training play a critical role in preparing a competent pharmacy workforce to meet the healthcare needs of the public. This study aimed to evaluate the effectiveness and feasibility of implementing a structured Continuing Professional Development (CPD) training programme for pharmacists in Kuwait. Twenty-one practicing pharmacists participated in the programme. This study evaluated the programme’s effectiveness and feasibility by analysing data from three sources: (1) two focus group interviews, (2) CPD records collected in May and November 2019, and (3) online survey responses collected at the middle and end of the programme. Findings from this study showed that implementing a structured CPD programme is feasible and could positively impact pharmacists’ practice. A guiding competency framework and continuous feedback from programme instructors added valuable support for pharmacists during the programme, and facilitated an impactful translation of education into practice. This study provides the first data on pharmacists’ CPD in Kuwait and serves as a starting point for future education plans, studies, and transformational actions pertaining to pharmacy workforce education and training.

## 1. Introduction

A competent pharmacy workforce is fundamental for ensuring the provision of high-quality healthcare services. Education and training play a critical role in preparing a competent pharmacy workforce to meet the healthcare needs of the public [1]. Maintaining competence also means that a pharmacy degree can no longer serve as an endpoint to professional training within the discipline of pharmacy. The need for continued learning after the completion of basic formal education, in which learning takes place over a lifetime and stretches beyond the classroom to point-of-care, has become imperative. Global and international initiatives support the concept of lifelong learning for pharmacists to maintain competence [2,3,4]. Several models, such as Continuing Education (CE) or Continuing Professional Development (CPD), have been adopted worldwide [5,6]. A main component of these models is the ability of pharmacists to identify and assess their own needs and to participate in an educational activity that would positively impact their practice. In the CE model, each learning activity is usually assigned a specific number of points or CE hours. Pharmacists’ level of engagement is often measured by the number of points or CE hours collected every year. This model may force some pharmacists to engage in non-preferred activities to accumulate the required number of credits, and the learning activities may not be tailored to meet the individual needs of pharmacists. CPD, on the other hand, includes but goes beyond CE. The CPD process is a continuous four-step cycle to reflect, plan, act, and evaluate, with each step documented throughout the process. With appropriate training and support, pharmacists can utilize a CPD approach for their life-long learning and professional development [7,8]. Pharmacists who regularly participated in CPD training reported that their perceptions of various aspects of their pharmacy practice improved as a result of their education activities more often compared to pharmacists who participated in traditional CE [9].

In 2016, the International Pharmaceutical Federation (FIP) announced 13 workforce developmental goals that were built together to establish the milestones for impactful global development for pharmacy education and to guide global, regional, and national transformations of the pharmacy workforce [10]. Moving from a global to a national level, foundation training, competency development, and CPD strategies workforce goals were identified as top priority goals for advancing the pharmacy workforce in Kuwait [11]. Moreover, the New Kuwait Vision 2035 recognises “improving the healthcare services” and “reforming the education system” as important pillars of the vision towards a prosperous and sustainable future for the country [12]. 

Kuwait has a population of around 4.1 million. The healthcare system in Kuwait provides primary, secondary, and tertiary healthcare services in both public (government) and private sectors, and is divided into six regional health areas: Capital (Al-Asema), Hawally, Ahmadi, Jahra, Farwania, and Al-Sabah. Out of the 4377 registered pharmacists in Kuwait, 1417 pharmacists work in the governmental sector at Ministry of Health (MoH) healthcare facilities. There is no re-licensure or revalidation process for pharmacists in the governmental sector. Governmental sector licenses expire when a pharmacist resigns/retires or ends his/her contract. In contrast, in the private sector, the pharmacy practice license needs to be renewed every five years for Kuwaiti pharmacists, and every two years for non-Kuwaiti pharmacists. Moreover, there are no regulations currently in place that mandate that pharmacists attend or keep a log of CE or CPD activities.

As in other Eastern Mediterranean Region (EMR) countries, in Kuwait, the primary role of pharmacists remains the traditional role of dispensing, supplying, and compounding medicines, as well as stock control and personnel management [13,14,15,16,17,18,19], with few structured clinical engagements and little advanced collaborative practice [20]. A previous study showed that pharmacists and CPD providers recognised the importance of life-long learning for pharmacists and acknowledged the current ad hoc nature of CPD opportunities in Kuwait [11]. In the same study, education providers also acknowledged the existing gap between initial education and actual practice, a gap that they believed could be filled by effective CPD strategies. Pharmacists, on the other hand, were found in need of early career maps and frameworks to support a seamless transition into early career practice. These frameworks were viewed as necessary to consolidate needs-based foundation training infrastructures for the novice workforce towards advanced practice. Although it is still not widely adopted, the Kuwait Foundation Competency Framework (KFCF) was developed to support pharmacists’ career progression and to act as a developmental tool that would help pharmacists in their life-long learning journey [21]. However, there is a growing need for evidence that provides insight into the infrastructures required to adopt the CPD model, which can become a base for the country’s strategic plans for workforce development, education, and training. 

In 2019, the Kuwait Ministry of Health (MoH) established a steering committee for the advancement of the pharmacy workforce and practice. One important objective in the committee mandate was to develop and implement a compulsory foundation training programme for newly employed pharmacists. The main aim of the foundation training programme is to provide competency-based training, with the goal of acquiring core foundation level competencies outlined in the Kuwait Foundation Competency Framework (KFCF) [21]. The programme also comprises a CPD model as a life-long learning approach, and utilises the KFCF as a supporting developmental tool for pharmacists. The overall goal of the CPD component of the programme is to help pharmacists to meaningfully engage in CPD and act differently in their practice. The integration of the KFCF would help pharmacists in the identification of learning needs by mapping their current performance with competencies and behavioural statements found in the KFCF. The utilization of the KFCF would also support the design and development of learning activities that meet participants’ learning needs. The CPD programme would help create change in the way pharmacists pursue continuing education (not just attending an educational activity, but rather learning, applying, and acting differently in real practice to create change and improvement in the services they provide), and the way education providers design and deliver CPD programmes (planned and structured rather than ad hoc and opportunistic) [11]. The programme and its elements and activities were planned to be flexible in a way that could engage pharmacists from different practice settings, including those in non-direct patient care and those in administrative positions. The programme used a flexible mode of delivery and interaction with participants, and included a diversity of topics. This was guided by previous literature that showed that flexibility in timing, diversity of topics, and variety in the mode of delivery may encourage and motivate pharmacists to participate in the programme and help them better apply the new knowledge, skills, or competencies in their real-life practice [22]. The programme was extended over a relatively long period of time to help pharmacists meaningfully engage in CPD and act differently in their practice. Spreading the programme over an 18-month period provided support for pharmacists while they adopt new behaviours. This also allowed pharmacists enough time to apply what they learned in their practice settings and to become familiar with the concept of CPD, apply the CPD cycle (along with application and evaluation steps), and create the change required according to their identified learning needs.

Before national implementation, a study that was intended to assess the feasibility and effectiveness of the CPD component of the foundation training programme was conducted. Assessing feasibility helps in determining whether the intervention is appropriate, relevant, and sustainable through focusing on general areas, such as acceptability, implementation, practicality, adaptation, and integration [23]. Effectiveness of a training intervention, on the other hand, can be measured by assessing the impact of the training on a trainee’s performance and behavior [24]. This study aimed to evaluate the effectiveness (in terms of application of the five-step CPD cycle and impact on practice) and feasibility (in terms of acceptability, adoption, and implementation) of the structured CPD training programme for pharmacists in Kuwait.

## 2. Methods

### 2.1. Study Design and Setting

This study was conducted using a mixed methods design. Mixed methods research has been widely used in education and health sciences [25]. A mixed methodological design has the strength of utilising both qualitative and quantitative research methods and minimising their limitations, as well as enhancing the validity of the findings [25,26]. The effectiveness of the programme was evaluated using quantitative (e.g., survey) and qualitative data (CPD record content analysis and focus group interviews), while the feasibility was evaluated using qualitative methods (focus group interviews and CPD records content analysis).

This study was conducted in the Capital health region in Kuwait. There are 28 primary care centres and one general hospital in the Capital health region, with around 151 pharmacists practicing in these healthcare facilities.

### 2.2. Participants and Intervention

**Figure 1:**
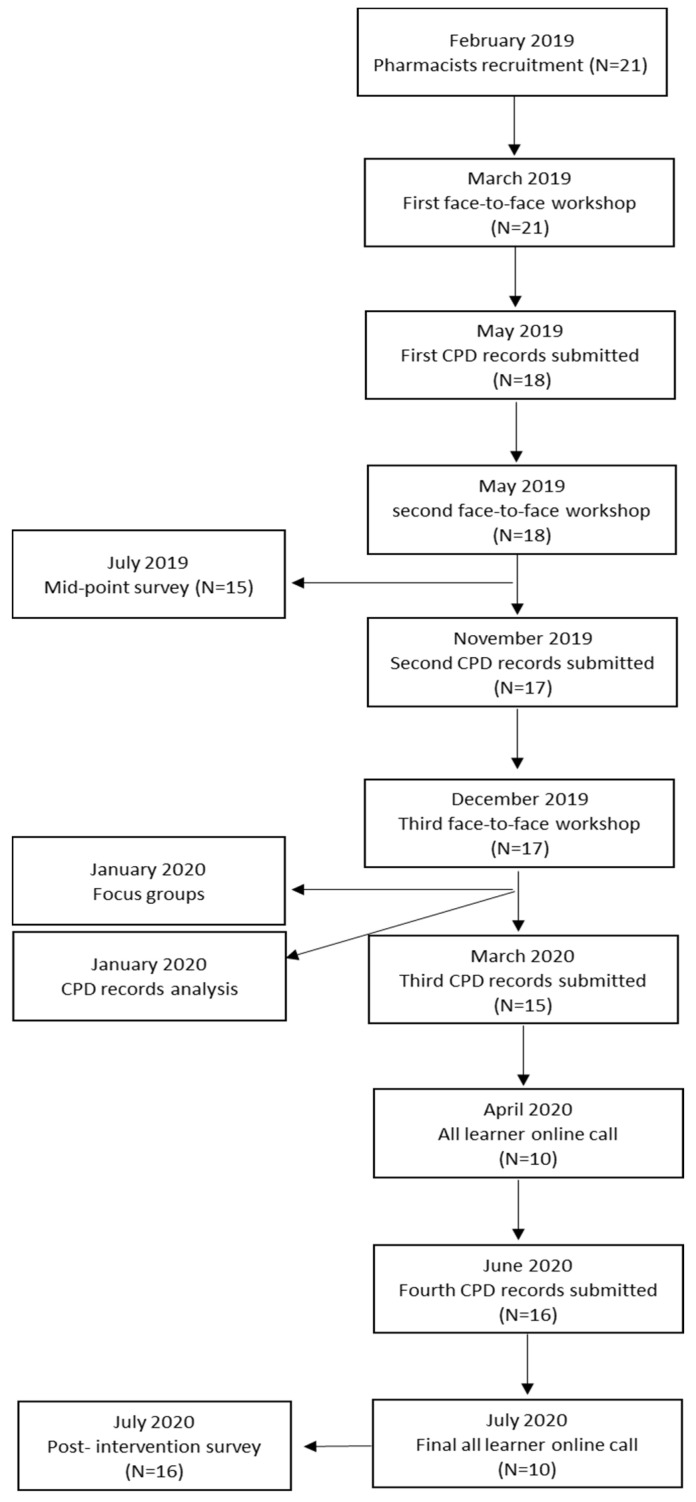
Programme timeline.

The programme consisted of three main components: (1) face-to-face workshops, (2) an online platform with resources related to CPD literature, and (3) CPD documentation and submission. Two instructors, who were also teaching staff members from the Faculty of Pharmacy at Kuwait University, delivered the workshops and reviewed all CPD documentation. The programme was started in March 2019 and was completed in July 2020. Each participant was required to attend three workshops, and to document and submit their CPD activities (Figure 1). Two additional online meetings were conducted to provide additional support to participants in April and July 2020 during the COVID-19 pandemic. The programme focused on generic skills important for life-long learning in general. The content of the programme is presented in Table 1. The programme also aimed to create a “training the trainer” opportunity in which participants were encouraged to share their learning with their peers and colleagues. The programme was supplemented by the KFCF to support participants in their professional development. The KFCF is an evidence-based tool that supplements training programmes at a foundation level, aids in the identification of learning needs, and supports the design and development of learning activities. The KFCF is comprised of 98 behavioural statements collected under 20 competency groups in four main clusters: (1) pharmaceutical public health competencies, (2) pharmaceutical care competencies, (3) organisation and management competencies, and (4) professional/personal competencies.

The details of the CPD programme were communicated to the primary care pharmacy manager and the director of the pharmacy department at the main general hospital in the Capital area. Both managers then nominated the participants. Participation in the programme was voluntary, and pharmacists could opt out of the programme at any time without explanation. Participants who completed all three components were eligible for a certificate of attendance issued jointly by Kuwait University and the Kuwait MoH. Participants documented their CPD using an electronic (Microsoft Word document) CPD record template (Supplementary File S1) and were required to send their records back as email attachments according to pre-specified submission deadlines. Written feedback on the submitted CPD records was provided to each participant with guidance for further improvement. One-on-one coaching sessions were also provided to participants upon their request. The programme instructors communicated with the participants through email, WhatsApp, and a private group on Microsoft SharePoint.

#### Ethical Consideration

Ethical approval for this study was obtained from the ethics committee at the Health Sciences Center, Kuwait University, and from the Ministry of Health ethics committee (2019/1227–2019/1228). Participants were required to complete the consent form before participating in the focus group.

### 2.3. Evaluation Methods Used

Programme evaluation was planned at different time points. The evaluation plan was comprised of focus group discussion, CPD record analysis, and an online survey. The focus groups and CPD records analysis were conducted at a mid-point of the programme (Jan 2020) to provide feedback for the instructors and to address any issues with the programme design or assessment.

### 2.4. Data Collection Procedures

#### 2.4.1. Focus Group Interviews

All participants were approached and those who agreed to participate in the focus groups were recruited. Two focus groups (each consisting of five pharmacists, an interviewer, and a moderator) were conducted in January 2020 to explore the perceptions of pharmacists who participated in the structured educational programme. A topic guide was developed by the research team and piloted with one participant to ensure clarity of questions and removal of any ambiguity. The topic guide included questions about participants’ experiences and their perceptions about the content and delivery of the programme. The questions were also related to their perceived barrier in pursuing CPD and their suggestion on how to spread the CPD concept. The data collected from the focus group interviews aimed to provide in-depth information about participants’ perceptions of how the programme impacted their practice and if the programme was effective in introducing the CPD concept. Moreover, the focus group aimed to fulfill the second aim of this study, in which participants would report their opinion on how feasible the progrmme was and if they would suggest any recommendation to facilitate national implementation.

#### 2.4.2. CPD Records Content Analysis

Content analysis was used to analyse the CPD records for the first two entries: May and November 2019. The CPD record analysis aimed to assess the effectiveness of the programme in participant adaptation and capability to follow the CPD process (e.g., feasibility). CPD records were anonymized by replacing the participant name with a code. Unique identifiers were used to match both May and November submissions to each participant. CPD records for the same participants were given the same identifier code.

#### 2.4.3. Survey

A link to the online survey was sent to all participants in July 2019 and July 2020. The survey was designed to assess the effect of the CPD programme on participants’ perceptions of factors related to pharmacy practice. The survey was adopted from McConnell et.al [9] and Dopp et al. [7]. In order to fulfill the aims of this study, the survey included statements about participants’ level of confidence and satisfaction with their practice compared to the past six months [9], their confidence with applying the five steps of the CPD cycle [7], their ability to use assessment tools (framework) for their professional development [9], their interaction with patients and initiation of changes in their workplace [9], and their perception towards the workshops in enhancing their professional knowledge, skills, and attitudes [9]. Participants were asked to rate their agreement with 15 statements using a five-point Likert scale (1 = strongly disagree, 2 = disagree, 3 = neutral, 4 = agree, and 5 = strongly agree). The survey was administered anonymously at two time points (mid-point and end of the programme). At the first time point, the survey was intended to provide feedback about participants’ perceptions on the impact of the programme on participants’ practices and their satisfaction with the overall structure of the programme (e.g., effectiveness). At the second time point, the survey aimed to inform a larger scale implementation on a national level (e.g., feasibility).

### 2.5. Data Analysis

Focus group audio records were transcribed verbatim. Textual data were analyzed manually using the thematic analysis method [27,28]. This involved six steps. 1—Familiarisation with the data: SA transcribed all interviews verbatim and repeatedly read the transcripts to have a general sense of the overall meaning of the data. 2—Identifying a conceptual framework: the process of developing framework categories was informed by emergent issues arising from the earlier familiarisation step. 3—Coding: SA applied the conceptual framework and systematically indexed all of the data in textual form by annotating the transcripts with codes and identifying any emergent codes. The first author (AA) checked them against the initially assigned codes to identify any disagreements and to further refine the codes. Discrepancies were discussed on several occasions. 4—Charting and reviewing themes: SA rearranged codes according to the appropriate themes to which they related. SA and AA discussed similar codes and codes that overlapped, and agreed upon a final set of themes. 5—Mapping and interpretation: in this step, a map of themes and the relation between them was produced. 6—Writing up the report. Although the previous steps were followed to ensure a systematic approach for data analysis, the process was iterative and involved a lot of back and forth between different ideas rather than a linear step approach. Several measures were undertaken to enhance the credibility (e.g., trustworthiness, representativeness, and robustness) of the qualitative data generated from this study. The credibility of qualitative data was maintained by continuously and thoroughly discussing data collection and the analysis processes with the research team. Moreover, the interview topic guide was pre-tested to confirm clarity and remove ambiguity. The same interviewer conducted the two focus group interviews to maintain consistency across groups and to minimise the variability that can occur when using multiple interviewers. Interviews were audio recorded and transcribed verbatim to help the researcher immerse in the data. The researchers, who were involved in focus group analysis, kept a reflection journal as a way to maintain trustworthiness and credibility of the methods used and to reduce researcher bias and subjectivity. The researchers’ reflective diary documented the researchers’ assumptions, beliefs, and bias that might have influenced the investigations and, consequently, data analysis. Writing notes and memos as part of the reflection journal also helped to record themes that needed further exploration.

**Figure 2:**
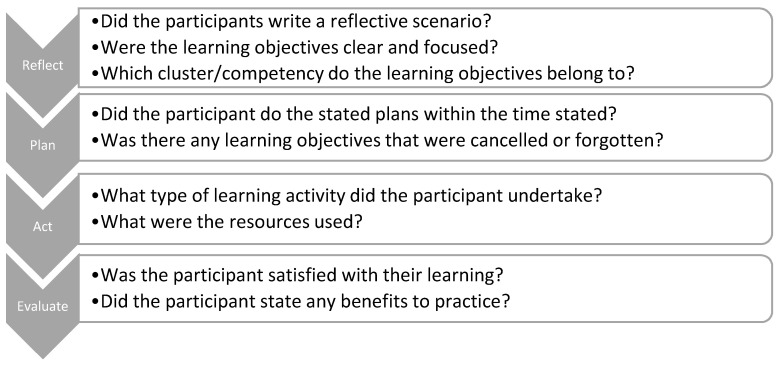
Framework used to analyse the CPD records.

Analysis of the CPD records was done systematically according to the framework presented in Figure 2. AA and AA analysed the CPD records independently, and discrepancies were discussed on several occasions. For the reflect step, numbers of participants who mentioned a reflective scenario were extracted. In addition, learning objectives listed in step 1, reflect, were mapped to KFCF clusters and competencies. Some participants stated the cluster they chose next to their learning objectives. These were taken as written. In step 2, plan, the numbers of learning activities listed were extracted, and activities that were not included in the action section were considered cancelled or forgotten. For the act step, the type of activities undertaken (e.g., discuss with other colleagues, teaching other colleagues, etc.) and the resources that participants used for their learning activities were extracted. Finally, for the evaluate step, participants’ level of satisfaction with their learning and if their CPD have benefited their workplace (e.g., patients’ satisfaction, benefits to colleagues, etc.) were extracted as documented by participants. These steps were followed for May entries and then repeated for November entries. Then, the improvement of each participant from May to November was compared according to the analytical framework to evaluate his/her documentation of each CPD step.

The survey data were exported to Microsoft Excel and analysed descriptively using numbers and frequencies. All data were analysed anonymously.

## 3. Results

Sixteen participants (out of 21 participants) completed all programme components and received a certificate of attendance (Table 2).

### 3.1. Focus Groups

Thematic analysis resulted in seven major themes describing the following progamme-related aspects: overall satisfaction, identifying and fulfilling learning needs, CPD documentation, communication and feedback, CPD submission, barriers to CPD, and support for a larger scale implementation.

#### 3.1.1. Theme 1: Overall Satisfaction

Participants were generally satisfied with the opportunity to participate in the programme: “This programme was overly exciting and enjoyable; instructors were helpful and always looking for the bright side of our work” (P1). They also commented that the programme provided a new concept and experiences that differ from other traditional continuous educational programmes that they had attended before. Moreover, participants reported that the diversity of workshop content and learning methods and flexibility of the programme kept them interested and keen to participate: “I think this programme is different because it is created by professional academics and presented in different creative styles” (P1). Participants liked the idea of meeting new colleagues and working as a team during workshop activities, and reported that this was supportive. Participants agreed that they felt self-satisfied as a result of this programme, pointing out that the feeling of achievement, whether on a personal or a professional level, was rewarding.

#### 3.1.2. Theme 2: Identifying and Fulfilling Learning Needs

Participants reported that every workshop’s content was useful, and each workshop built on the previous one. They also identified areas for improvement and were able to work on the challenges they faced in systematic and organized approaches: “This is a different experience that showed me to how to identify my weakness and provided me with methods to solve them…” (P6). Participants mentioned that the workshops helped them identify the knowledge and skills they needed for their personal and professional development.

#### 3.1.3. Theme 3: CPD Documentation

Participants described the template used for the CPD documentation as simple and easy to follow: “First of all, it helped us discover our weaknesses and document it in a report in a neat manner so that we know our mistakes and how we can develop” (P4). However, certain parts of the template needed more clarification, as one participant explained: “[the CPD template’s] questions are easy, but the tables are difficult and confusing…” (P9). They also suggested improving the template design by providing more space and more instructions. Providing some examples on how to fill in the tables and other sections of the template, as well as some simple tips to be used as a guidance for future submissions, was deemed important.

#### 3.1.4. Theme 4: Communication and Feedback

After each CPD record submission, the programme instructors provided each participant with verbal and written feedback. Participants reported that they appreciated the instructors’ feedback and the ongoing communication with them. They found the feedback helpful and constructive: “Each feedback developed me in different aspects…” (P5). They felt empowered to work harder and achieve more, and they were encouraged “to go and discuss with the instructors in a one-to-one coaching session if there is something, we didn’t understand…” (P4). One participant mentioned that she was still not confident enough to perform a full CPD cycle, however, she found the instructor’s comments guided her to improve her CPD skills. Participants also mentioned that the written feedback needed to be more specific and clearer. Different platforms of communication were used, including Microsoft SharePoint, E-mail, and WhatsApp. However, participants generally were in agreement that WhatsApp was the best communication channel.

#### 3.1.5. Theme 5: CPD Submission

Participants found that the deadline time was suitable for them, as they were newly introduced to the concept and therefore needed sufficient time. One participant thought that the “deadline time was perfect**…**” (P8). Overall, participants agreed that deadlines were useful, as they forced them to organise their plans efficiently. However, there was a suggestion that submitting the required number of CPD records by the end of year would be more suitable for them: “It is better to avoid deadlines because each cycle differs than others…” (P6).

#### 3.1.6. Theme 6: Barriers to CPD

Through the discussion about obstacles they faced during this programme, lack of time to work on CPDs was a big issue. Participants suggested that to resolve this issue, either the total number of CPD cycles should be reduced or they should have dedicated time during work hours for the CPD activities. According to participants, lack of support and “lack of appreciation” (P2) from their managers and colleagues was frustrating and it "makes us hard to continue working” (P2). Some of their colleagues communicated their resentment when the participants left their work to attend the workshops. One participant suggested to select one permanent location to hold the workshops.

#### 3.1.7. Theme 7: Support for a Larger Scale Implementation

To stay on top of the CPD requirements, participants suggested that they would need more support. They found that support provided by the programme instructors was not enough. Participants had several suggestions to improve the CPD programme and to make larger scale implementation more feasible (Table 3). More support from higher authorities was needed. More appreciation from their managers and colleagues was considered an important factor. They also believed that their participation in this programme should be counted with a certain number of continuing education (CE) points linked to their promotion: “I wish when I submit my CPD records I get CME points” (P2). It was suggested that to implement the CPD concept on a national level, an online platform should be developed to ensure sustainability and consistency. They also suggested to increase the number of reviewers: “the number of the trainers is considered a small number, we need to have more trainers in order to increase the number of attendees” (P2), and to make sure that they have the ability to evaluate specialised CPD records. One participant proposed the inclusion of the CPD concept in the Faculty of Pharmacy curriculum. Other participants went further to demand that this programme should be mandatory and linked with promotion. On the other hand, some participants thought that voluntary participation would be enough if the programme became recognised nationally by public and private employers.

### 3.2. Content Analysis of CPD Record

A total of 35 CPD records were analysed. These records were collected in May 2019 (first entry, n = 18) and November 2019 (second entry, n = 17). The record analysis focused on the five-step CPD cycle (Table 4) and participants’ utilization of the KFCF in their professional development (Table 5).

#### 3.2.1. Plan

In the plan section, participant documentation showed that they managed to complete their plans in the time chosen by each of them. However, in both entries, some CPD records showed some plans that had not been done at all, even cancelled by the participant, or forgotten. A plan to “Learn about major depressive disorder’s pathophysiology” (P5) was not followed through and there was no explanation provided.

#### 3.2.2. Act

The action step was straightforward for some participants. The CPD records showed that participants were able to state a clear description of the CPD activity they planned to pursue. The most common types of learning activities were discussion with colleagues and delivering lectures to colleagues. On the other hand, participants used different resources to achieve their plans, e.g., participant 9 stated in a November entry that she/he attended a lecture, read a chapter in a textbook, and used different online resources.

#### 3.2.3. Evaluate

For the evaluate section, participants showed a clearer evaluation of the plan and actions in their November CPD records as opposed to their May CPD records. The following are two examples of two records submitted in different times by participant 5: “I feel more confident about advising patients with asthma” (submitted in May), and “met the target in completing and submitting incidence reports” (submitted in November). It was evident that the feedback given by the instructors for each record had an impact on developing the participants’ skills. All participants reported that they achieved their learning objectives. Some participants, however, needed more support in this section, as there was some confusion about how to evaluate the impact of their learning on patient care or their workplace.

#### 3.2.4. KFCF

As the KFCF was used as a supportive developmental tool in the programme, participants reported the focus of their CPD according to KFCF clusters and behaviours. Participants who wrote the clusters were found to have written clear and focused objectives, such as “to differentiate between the serious side effects that warfarin may cause and when to refer to the doctor” (P11), compared to those who only listed the learning objectives, such as “Familiarize myself with all antibiotics available in MOH that can be taken once daily orally” (P13). They also wrote more details about their planned activities and had achieved their goals or at least found the way to do so by the deadline. It was found that in their first submission, 44.4% (n = 8) of participants focused on cluster 2 (Pharmaceutical Care Competencies), while in the second submission, the majority of participants shifted to cluster 1 and cluster 4 (Table 4).

### 3.3. Survey

Response rates to the online survey were 71.4% (n = 15) and 76.2% (n = 16) for the middle of the programme (Jul 2019) and end of the programme (Jul 2020), respectively. Table 6 shows participants’ responses to the survey. The participants, on average, agreed or mostly agreed with a positive impact of the programme on their performance in the workplace, as well as their satisfaction with workshop content and activities. This was found to be in line with the results obtained from the focus group interviews.

## 4. Discussion

This study is, to our knowledge, the first study in Kuwait and the Eastern Mediterranean Region (EMR) that investigates the feasibility of a structured CPD programme for pharmacists. With limited evidence related to pharmacists’ professional development in the EMR, this study fills an important gap in the literature and adds valuable insights that would guide educators, policymakers, and leaders in other countries towards advancing pharmacy professional development in their local context. Our findings suggest that implementing a structured CPD programme is feasible and effective, as it positively impacted pharmacists’ practice. The findings from the focus group interview, the CPD records, and the surveys showed that participants in this study reported that they were able to adopt and implement the CPD model in their daily practice. Participants reported that the programme was effective in enhancing their ability to better interact with patients and other healthcare providers, and increased the depth of patient counselling (e.g., impact on practice). A guiding competency framework and continuous feedback from programme instructors added valuable support for pharmacists during the programme and facilitated an impactful translation of education into practice.

Findings from the present study suggest that the programme proved successful in introducing the CPD concept to participants and helped them apply it in their daily practice. In their CPD records and results from the surveys, participants reported that they were able to apply all five steps of the CPD cycle, succeeded in initiating changes in their practice, and were able to apply learning to work. This is consistent with previous studies that implemented structured educational programmes to familiarise pharmacists with the CPD model and support them to adopt the concept to maintain their competence [7,8,9,29,30].

Findings from the focus groups showed that the programme design, content, and delivery methods were perceived as satisfactory. Participants appreciated the interactive portion of the workshops and were willing to adopt CPD as a new method to proactively direct their learning. Similar findings were reported by Tofade et al. [29], where active learning exercises, delivery style, and combination of self-study and active live learning for CPD programmes were very effective in motivating participants to implement CPD in their practice.

In the present study, it was found that participants did not often reflect upon their performance, however, those who had reflected on their performance were more precise and focused on what they wanted to achieve. The CPD record analysis showed that the number of participants who stated a reflective scenario increased from the first entry to the second entry, and those who performed reflective practice were more likely to achieve their stated learning objectives. Similar findings were reported in a previous study, in which participants generally did not often reflect and pre-plan their learning activities, however, when learning activities were planned, it appears that they had a higher tendency to accomplish them [29]. This could be overcome by adding a section in the CPD template for participants to write their reflection in, as the CPD template used in the present programme had no designated space for a reflection scenario, and this may have misled participants to jump directly to stating their learning needs and objectives. Evaluation of CPD activities was found to be challenging for pharmacists, as was also reported by Tofade et al. [29]. Participants of this study reported that filling the CPD template required time and sometimes needed more clarification on how to fill certain parts of the template. This is consistent with previous studies that reported pharmacists were found to have difficulties in identifying their training and learning needs or in evaluating their participation in CPD activities [31,32,33]. However, participants of this study reported during the focus group interviews that meeting with new colleagues during the learning activities and sharing their CPD experiences with their peers during face-to-face workshops, in addition to the continuous feedback from programme instructors, was helpful in improving subsequent submissions in the present study.

The survey showed that participants agreed that using a structured self-assessment tool (e.g., KFCF) helped them identify practice strengths and areas for improvement. This was found to be consistent with a previous study that used assessment tools to support pharmacists applying the CPD concept in their daily practice [7]. Similar to McConnell et al. [9], participants in the present study agreed that they reinforced learning through practice, applied learning to work, and initiated more changes in work or practice as a result of the education activities.

Participant focus groups, CPD records, and online surveys showed that the programme positively impacted their practice. Participants reported that they initiated more changes in their workplace and were able to better interact with patients and other healthcare providers. However, more support from colleagues and higher authorities was needed, as reported during the focus group interviews. Similar studies also showed that peer, manager, and employer support were needed to adopt a CPD approach in the workplace [22,33,34,35]. The steering committee in the MoH is working on advocating for the new foundation training programme through communication channels with pharmaceutical directors of each health region, in addition to other key stakeholders, which will help provide adequate support for future programme participants.

### 4.1. Implications for Development by Other Nations

This study attempted to investigate the feasibility and effectiveness of a structured CPD educational programme for pharmacists in Kuwait. The mixed-methods design helped provide a comprehensive evaluation of the programme, and provided insights on the required infrastructure to implement such a programme on a national level. The infrastructure used in the present study included (1) face-to-face workshops that were delivered during “protected times” for all pharmacists joining the programme, (2) workshop content that aimed to provide pharmacists with the essential knowledge and skills to identify their learning needs and act upon them, (3) flexible mode of delivery, including face-to-face and online training, material for self-study, and online resources, which helped sustain the programme during the COVID-19 pandemic, (4) using the KFCF as a developmental tool to help pharmacists reflect on their current practice and identify learning gaps, (5) written and verbal feedback and continuous communication between participants and instructors via different platforms, and (6) spreading the CPD programme over a period of 18 months to ensure that proper support was provided for pharmacists while they adopted new behaviours, which allowed pharmacists enough time to apply what they learned in their practice settings, become familiar with the concept of CPD, and apply the CPD cycle (along with application and evaluation steps). However, effective and clear policies and regulations that govern pharmacy CPD and pharmacy practice in Kuwait are currently lacking. Policies and regulations are an important component for the sustainability of the CPD programme. Effective workforce development strategies require policy and regulation formation. The steering committee at MoH, therefore, is currently planning for a wider CPD policy that would be applied on a national level.

### 4.2. Limitations

This study has some limitations. First, conducting focus groups may be subjected to social desirability bias, where interviewees may illustrate or report good behaviours or experiences to please the interviewer or other participants [36]. The interviewer in this study, however, frequently prompted participants during each interview to better understand their responses and behaviours in different situations and experiences. Another limitation is that the long-term impact of the CPD programme on the participants’ practice was not addressed. However, future studies may aim to assess the impact of the programme on participants’ performance and the sustainability of their CPD behaviours. Finally, findings from this study will inform nation-wide CPD strategies. Therefore, future research could explore and evaluate the real experiences of pharmacists in regard to complex issues related to their professional development, and provide a foundation for ongoing research into pharmacy workforce development.

## 5. Conclusions

This is a novel study that provided an original, insightful examination of pharmacy professional development in Kuwait. The identified infrastructure provides a roadmap for pharmacists’ education and professional development that could be adapted by other nations. This study provides the first data on pharmacists’ CPD in Kuwait and serves as a starting point for future education plans, studies, and transformational actions for pharmacy workforce education in Kuwait and EMR countries.

## Figures and Tables

**Table 1:** Workshop content.

Workshop Outline		Mode/Duration
Workshop 1	Day 1: CPD, CE definitions and evidence CPD documentation The professional development models Kuwait Foundation Competency Framework (KFCF) Day 1 focus: Cluster 1: Pharmaceutical public health cluster Day 1 focus: Cluster 2: Pharmaceutical care cluster Day 2: Change management and change adaptation Day 2 focus: Cluster 3: Management and organisation Day 2 focus: Cluster 4: Professional /personal Plan personal CPD cycle: work on individual CPD cycle Day 3: Participants’ presentations of their CPD cycles Discuss plans to increase awareness of CPD in your workplace	Face-to-face 5 h/day
Workshop 2	Feedback on previous submission Reflections models (reflection step) How to write a SMART goal? (plan step) What measurements are important (evaluation step)	Face-to-face 5 h/day
Workshop 3	Feedback on previous submission What is learning/motivation to life-long learning CPD plan and evaluation research	Face-to-face 5 h/day
All learner call 1	Feedback on previous submission COVID-19: Times of uncertainty Moving away from your comfort zone	Online using Zoom 1.5 h
All learner call 2	Feedback on previous submissions Findings from programme evaluation What is next?	Online using Zoom 1.5 h

**Table 2:** Participants’ demographic characteristics (N = 21).

Demographic Characteristics	N (%)
**Gender**	
Male	3 (14)
Female	18 (86)
**Nationality**	
Kuwaiti	19 (90)
Non-Kuwaiti	2 (10)
**Practice setting**	
Primary care	19 (90)
Hospital	2 (10)
**Years in practice**	
0–5 years	15 (71)
6–10 years	3 (14.5)
>10 years	3 (14.5)

**Table 3:** Participants’ suggestions to improve the CPD programme and to help implement the concept of CPD nationally.

*“Develop an official online database for CPD records” (P4).*
*“Include the CPD programme as a mandatory course in the pharmacy college curriculum” (P2).*
*“Simplify the CPD template design” (P7).*
*“Provide model examples of completed CPD forms” (P7).*
*“Increase the number of CPD records reviewers or instructors with variable capabilities” (P2).*
*“Link the number of CPD cycles performed per year to career promotion and CME points” (P2).*
*“Show off the added value and privilege of participating in the CPD programme” (P7).*
*“More clarified regulations regarding the late submissions during the CPD course” (P7).*

**Table 4:** Analysis of the five steps of the CPD cycle.

Steps of the CPD Cycle	1st Submission May 2019	2nd Submission November 2019
	N (%)	N (%)
**Reflect**		
*Stated a reflective scenario*		
Yes	8 (44)	13 (76)
No	10 (56)	4 (24)
**Plan**		
Managed to complete their plans *		
Yes	16 (89)	17 (100)
No	4 (21)	0 (0)
**Act**		
*Stated a clear description of the CPD activity*		
Yes	10 (56)	12 (71)
No	8 (44)	5 (29)
Most common types of learning activities **		
Discussion with colleagues	12 (67)	9 (53)
Giving lectures	5 (28)	9 (53)
**Evaluate**		
*Satisfaction with achievement of learning objectives*		
Satisfied	18 (100)	17 (100)
Not satisfied	0 (0)	0 (0)

* Numbers of May entries exceeded 100% because some participants managed to achieve some of the stated objectives, but not all of them. ** Numbers may not add to 100% as some participants mentioned more than one type of activity.

**Table 5:** Focus of CPD records according to the KFCF clusters.

KFCF Cluster	1st Submission May 2019	2nd Submission November 2019
	N (%) *	N (%) *
Cluster 1: Pharmaceutical public health competencies	7 (38.9%)	7 (41.2%)
Cluster 2: Pharmaceutical care competencies	8 (44.4%)	2 (11.8%)
Cluster 3: Organization and management Competencies	5 (27.8%)	5 (29.4%)
Cluster 4: Professional/Personal Competencies	6 (33.3%)	7 (41.2%)

* Totals may not add up to 100% as some CPD records covered more than one cluster.

**Table 6:** The median and range of responses to the evaluation of the programme in 2019 and 2020.

Statement	July 2019 (n = 15)	July 2020 (n = 16)
	Median (Range) *	Median (Range) *
1. I feel more self-confident in my professional practice compared with 6 months ago	4 (3–5)	4 (3–5)
2. I have Higher level of job satisfaction compared with 6 months ago	4 (3–5)	4 (3–5)
3. I am confident in my ability/skill to identify my learning needs related to my work or professional practice	4 (4–5)	5 (4–5)
4. I am confident in my ability/skill to plan my learning and professional development	4 (4–5)	4 (4–5)
5. I am confident in my ability/skill to evaluate the impact or outcomes of my learning	4 (3–5)	4 (3–5)
6. I can use structured self-assessment tools (frameworks) related to my work or professional practice to help me identify my practice strengths and/or areas for improvement	4 (3–5)	5 (2–5)
7. I feel better able to interact with other health-care providers	4 (3–5)	4 (3–5)
8. I feel better able to answer patient questions	4 (3–5)	4 (3–5)
9. I increased the depth or level of patient counselling	4 (3–5)	4 (4–5)
10. I Initiated more changes in my work or practice	4 (4–5)	4 (3–5)
11. I’m able to reinforce learning through practice	4 (3–5)	4 (4–5)
12. I’m able to apply learning to work	4 (3–5)	5 (4–5)
13. The education activities during the workshops enhanced my professional knowledge	4 (4–5)	4 (3–5)
14. The education activities during the workshops enhanced my professional skills	5 (4–5)	5 (4–5)
15. The education activities during the workshops enhanced my professional attitudes and values	5 (4–5)	5 (4–5)

* 1 = strongly disagree, 2 = disagree, 3 = neutral, 4 = agree, and 5 = strongly agree.

## Supplementary Materials

The following are available online at https://www.mdpi.com/2226-4787/8/4/207/s1, File S1: CPD record.
