# Supplementary material for: The Development and Evaluation of a Structured Continuing Professional Development Programme for Pharmacists in Kuwait: A Feasibility Study
Source: Pharmacy (Basel). 2020 Nov 5;8(4):207. doi: 10.3390/pharmacy8040207 (PMC7712550; doi:10.3390/pharmacy8040207)
Supplement: Supplementary file 1 [file pharmacy-08-00207-s001.pdf]

My CPD record

CPD encompasses lifelong learning with a view to attaining consistent performance within the scope of practice

3/17/2019

Name:

Year in practice:

Practice site:

Date:

Reflect

- Reflect: Areas I have identified requiring professional development
    - Identify and describe the specific skills or competency to improve or develop based on the evaluation of your practice against behaviours in the KFCF
      To help you identify those needs ask yourself the following questions:
        - What do I need to know/ to be able to do?
        - What knowledge/skills/competencies would I want to develop/improve?
        - What situation happened that was difficult for me to act with or situations that I would like to act differently on it?
        - What areas of improvement does my supervisor/manager/head of department recommends to improve my practice?

**Learning objectives**<br><br>***(List of relevant skills/knowledge/competencies that I intend to improve or develop)***	**Rank them according to the priority**<br><br>(learning objective number)

Plan

- Plan: actions that I will do to meet my professional development needs.
  - Ask yourself the following questions?
    - What I'm going to do to achieve my learning objectives?
    - How can I learn and improve my knowledge/skills?
    - What are the activities I intend to undertake to meet my learning objectives**?
    - When I'm going to start and complete these activities?

Identified learning objective number	Planned activity	Goal start date

** learning activity types:

1. Work-based learning:	2. Professional activity:	3. Formal/educational:	4. Self-directed learning:
a) Case studies<br>b) Learning by doing and practice<br>c) Coaching from others<br>d) Discussions with colleagues<br>e) Involvement in wider work of employer (for example, being a representative on a committee)<br>f) Journal club<br>g) Supervising staff or students<br>h) Filling in self-assessment questionnaires<br>i) Project work or project management	a) Lecturing or teaching<br>b) Organising journal clubs or other specialist groups<br>c) Giving presentations at conferences<br>d) Supervising research	a) Academic Courses<br>b) Research<br>c) Attending conferences<br>d) Writing articles or papers<br>e) Going to seminars<br>f) Distance learning	a) Reading journals/articles<br>b) Reviewing books or articles<br>c) Updating knowledge through the internet

Act

- Action (do): to describe a range of development actions intended to meet your learning objectives.
    - Ask yourself the following questions:
        - What I have done to achieve my goals?
        - What are the title and the content of my activities?
        - What type of activities did I use?
        - When did I start and finish the activities?

Identified learning objective number	Type of CPD activity	Title of CPD activity with brief description	Actual start date<br>Actual finish data
			Start:<br>Finish:
			Start:<br>Finish:
			Start:<br>Finish:

Evaluate

- Evaluate: to demonstrate that you have evaluated the learning outcomes through completing these actions:
  - Ask yourself the following questions:
    - What did I learn through completing the activity?
    - How have my skills and knowledge improved or developed?
    - How does the activity contributes to the quality of my practice?
    - How does the activity benefits the patient?
    - Did the learning activity help me meet my learning objectives?

Learning activity number	Learning outcome from the activity	Were your learning needs met?<br>1. Fully<br>2. Partially<br>3. Not at all	If not fully met, what will you do differently in future?	Identified further needs as a result of the learning experience
